# Association of Overweight and Elevation with Chronic Knee and Low Back Pain: A Cross-Sectional Study

**DOI:** 10.3390/ijerph110404417

**Published:** 2014-04-22

**Authors:** Tsuyoshi Hamano, Masamitsu Kamada, Jun Kitayuguchi, Kristina Sundquist, Jan Sundquist, Kuninori Shiwaku

**Affiliations:** 1 Center for Community-based Health Research and Education (COHRE), Organization for the Promotion of Project Research, Shimane University, 223-8 Enya-cho, Izumo, Shimane 693-8501, Japan; 2 Department of Health Promotion and Exercise, National Institute of Health and Nutrition, 1-23-1 Toyama, Shinjuku-ku, Tokyo 162-8636, Japan; E-Mail: kamada@gakushikai.jp; 3 Division of Preventive Medicine, Brigham and Women’s Hospital, Harvard Medical School, 900 Commonwealth Avenue East, Boston, MA 02215, USA; 4 Japan Society for the Promotion of Science, 5-3-1, Kojimachi, Chiyoda-ku, Tokyo 102-0083, Japan; 5 Physical Education and Medicine Research Center UNNAN, 1212-3 Mitoya, Mitoya, Unnan, Shimane 690-2404, Japan; E-Mail: junk_907@yahoo.co.jp; 6 Center for Primary Health Care Research, Lund University, Clinical Research Centre (CRC), Floor 11, Building 28, Jan Waldenströms Gata 35, Skåne University Hospital, SE-205 02 Malmö, Sweden; E-Mails: kristina.sundquist@med.lu.se (K.S.); jan.sundquist@med.lu.se (J.S.); 7 Stanford Prevention Research Center, Stanford University, Medical School Office Building (MSOB), 251 Campus Drive, MC 5411, Stanford, CA 94305, USA; 8 Department of Environmental and Preventive Medicine, Shimane University School of Medicine, 89-1 Enya-cho, Izumo, Shimane 693-8501, Japan; E-Mail: shiwaku@jn.shimane-u.ac.jp

**Keywords:** physical environment, overweight, chronic knee pain, chronic low back pain

## Abstract

It is known that overweight is associated with chronic knee pain (CKP) and chronic low back pain (CLBP). Several risk factors for these conditions have been postulated, including age, sex, overweight, occupation, and socioeconomic factors. In addition, physical environment has been studied as a potential risk factor in recent years. However, the associations of overweight and physical environment with CKP and CLBP remains unclear. The aim of this study conducted in a rural mountainous region was to examine whether overweight individuals living at higher elevations have an increased probability of experiencing CKP and CLBP. In 2009, we conducted a mail survey with a random sample aged between 40 to 79 years. Questionnaires were sent to 6,000 individuals and a total of 4,559 individuals responded to this survey. After excluding the respondents with missing data, we conducted a logistic regression analysis of the data for 3,109 individuals. There was statistically significantly higher adjusted odds ratio (aOR) of CKP for those who were overweight living at low elevation (aOR = 1.90, 95% CI = 1.21–2.98), moderate elevation (aOR = 1.73, 95% CI = 1.05–2.87), and high elevation (aOR = 2.13, 95% CI = 1.31–3.46) than those who were not overweight living at low elevation. However, similar patterns were not observed for CLBP. Our results show that specific overweight–elevation associations were observed for CKP in a rural mountainous region.

## 1. Introduction

Chronic musculoskeletal pain in the knees and the low back is a common cause of reduced physical function and quality of life [1,2]. A national survey in Japan revealed that 4.3% of men and 7.7% of women reported pain in their limbs, and 8.7% of men and 11.7% of women reported low back pain [3]. These values are expected to increase as the population ages not only in Japan but also in other countries. Several risk factors for these conditions have been postulated, including age, sex, overweight, occupation, and socioeconomic factors [4,5,6]. Moreover, it is also important to note that geographical differences has been reported [7,8,9,10].

During the past decades, there has been growing interest in urban-rural differences. For example, a recent research found that the odds ratios of knee pain and lumbar pain were significantly higher in rural areas than in urban areas [10]. Another study suggested that the odds ratio of knee pain was higher in mountainous areas than in urban areas [7]. Therefore, considering the geographic area-oriented prevention strategy for musculoskeletal pain is needed, and exploring specific risk factors could aid in planning community-based approaches.

It is known that overweight causes musculoskeletal pain through increased strain on the joints during various activities [11]. The effect of overweight on musculoskeletal pain would be varied by physical environment. For example, previous study conducted in a mountainous area found that participants who felt steep slopes around their residence were more likely to report knee pain due to increasing mechanical stress [12]. Another study also suggested that physical environment related to musculoskeletal overload could be determinants in shaping the differences among geographic areas [9]. 

The nature of physical environment, *i.e.*, steep slopes around residence, is complex and consensus has not yet been established as to which measurement is the most accurate. Considering the context of Japan, elevation would be one of the proxies to measure for walking steep slopes in daily activities. This is why hilly and mountainous areas occupy approximately 70% and these areas are defined as those from outer plains to the mountains [13]. In the present study, it is hypothesized that overweight individuals living at higher elevations would have an increased probability of experiencing chronic knee pain (CKP) and chronic low back pain (CLBP). However, none of the studies have tested this hypothesis. The aim of this study was to examine the associations of BMI and elevation with CKP and CLBP in a rural mountainous region.

## 2. Methods

### 2.1. Participants

This is a cross-sectional study conducted in a rural mountainous region in Japan. In 2009, we conducted a mail survey with a random sample aged between 40 to 79 years. Those who stayed at care facilities, required long-term care, or could not complete the questionnaires by themselves due to disability were excluded from this study. Questionnaires were sent to 6,000 individuals randomly selected from the city registry in Unnan City. The total population of Unnan city was 43,520 at the time of this survey. Reminders were sent to the people who did not return the questionnaire within two weeks. A total of 4,559 individuals (76.0%) responded to this survey. The research ethics committee of the Physical Education and Medicine Research Centre UNNAN approved this study, and written informed consent was obtained from the respondents.

### 2.2. Measurements

#### 2.2.1. Chronic Musculoskeletal Pain

CKP and CLBP were assessed by a self-reported questionnaire that include questions similar to those in the Knee Pain Screening Tool (KNEST), except for the questions about use of health services (not examined in this study) [14,15]. The KNEST was previously developed to screen and identify individuals who have a knee pain in a general population. CLBP and CKP were defined as current pain, *i.e.*, episodes of pain at the time of the questionnaire that had lasted longer than 3 months in the past 1 year [16]. We assessed the test-retest reliability of CLBP and CKP by mailing the questionnaire twice to 500 randomly-selected adults aged 40–84 years, separated by an interval of 10 days. These were individuals living in Unnan city who were not invited to participate in the main survey. Evaluating the 206 respondents (mean and standard deviation of age = 63.4 and 11.9 years; women = 51.4%) to both questionnaires, we observed moderate reliability (Cohen’s kappa = 0.49 for CLBP and 0.72 for CKP).

#### 2.2.2. Elevation

GIS were used to estimate elevation on the basis of the individual’s address and for database queries. We used the ArcGIS 10.0 software developed by Environmental Systems Research Institute (Redlands, CA, USA). The elevation height above the mean sea level for each participant was assessed by the ready-to-use dataset of digital elevation models for ArcGIS. Participants were divided into three groups on the basis of the tertile value of elevation: 0–49.0 m = low elevation, 50.0–70.0 m = moderate elevation, and 71.0–468.0 m = high elevation.

#### 2.2.3. Other Measures

Age and sex were derived from the city registry and other variables were collected from the self-reported questionnaires: body mass index (BMI) was calculated using self-reported weight and height, engaged in farming (yes or no), educational attainment (years), self-rated health (excellent; good; fair; poor) [17], depressive symptom (yes or no) [18], history of chronic disease (hypertension, hyperlipidaemia, diabetes, hyperuricaemia, cerebrovascular disease, heart disease, kidney and urologic diseases, liver disease, gastrointestinal disease, endocrine disease, and/or cancer), history of chronic musculoskeletal disorder, and moderate-to-vigorous physical activity (MVPA) measured by the International Physical Activity Questionnaire [19,20].

### 2.3. Statistical Analysis

The analyses were restricted to the respondents who answered chronic musculoskeletal pain, weight, height and covariates completely. After excluding the respondents with missing data, we conducted a logistic regression analysis of the data for 3,109 individuals. A multivariable logistic regression model was used to derive adjusted odds ratio (aOR) for CKP and CLBP. Overweight-elevation association was categorized into the following six groups for the analysis: (1) participants with BMI < 25.0 and living at low elevation; (2) participants with BMI < 25.0 and living at moderate elevation; (3) participants with BMI < 25.0 and living at high elevation; (4) participants with BMI ≥ 25.0 and living at low elevation; (5) participants with BMI ≥ 25.0 and living at moderate elevation; (6) participants with BMI ≥ 25.0 and living at high elevation. *P*-values less than 0.05 were considered statistically significant. All statistical analyses were performed using SPSS for Windows (SPSS, Tokyo, Japan).

## 3. Results

The characteristics of study participants are shown in Table 1. The mean age (S.D.) was 59.0 (10.3) and 47.9% of the participants were men. The mean years (S.D.) of educational attainment was 11.7 (2.3). Almost 10% of participants reported CKP and 14.0% of participants reported CLBP. Almost half of the participants engaged in farming (47.1%), physically active (54.1%) and felt depressed (49.3%). Approximately 20% of the participants were overweight and 16.1% of the participants reported their subjective health condition as poor or fair. In addition, 457 (14.7%) of the participants had chronic musculoskeletal disorders history and 1,877 (60.4%) of the participants had chronic diseases history. Of the 3,109 individuals, 28.3% of the participants were not overweight and living at lower elevation. 

Table 2 shows the results of multivariable logistic regression analysis. For CKP, participants who were overweight had statistically significantly higher aORs (aOR = 2.05, 95% CI = 1.55–2.71) than those who were not overweight. There were no statistically significantly associations between elevation and CKP (moderate elevation aOR = 0.92, 95% CI = 0.68–1.24; high elevation aOR = 0.94, 95% CI = 0.69–1.29). For CLBP, participants who were overweight had not statistically significantly higher aORs (aOR = 1.23, 95% CI = 0.95–1.59) than those who were not overweight. There were no statistically significantly associations between elevation and CLBP (moderate elevation aOR = 0.82, 95% CI = 0.63–1.07; high elevation aOR = 1.03, 95% CI = 0.79–1.34).

**Table 1:** Characteristics of the participants.

Variables	Number of Participants	(%) or Mean (S.D.)
**Chronic knee pain, (%)**	321	(10.3)
**Chronic low back pain, (%)**	435	(14.0)
**Age, years (S.D.)**	3,109	59.0 (10.3)
**Sex**		
Men, (%)	1,488	(47.9)
Women, (%)	1,621	(52.1)
**Educational attainment, years (S.D.)**	3,109	11.7 (2.3)
**Engaged in farming, (%)**	1,464	(47.1)
**Self-rated health**		
Excellent/good, (%)	2,607	(83.9)
Fair/poor, (%)	502	(16.1)
**Depressive symptom, (%)**	1,534	(49.3)
**Chronic musculoskeletal disorders history, (%)**	457	(14.7)
**Chronic diseases history, (%)**	1,877	(60.4)
**MVPA**		
Active (≥ 150 min/week), (%)	1,681	(54.1)
Inactive (< 150 min/week), (%)	1,428	(45.9)
**BMI**		
<25.0, (%)	2,522	(81.1)
≥25.0, (%)	587	(18.9)
**Elevation**		
Low, (%)	1,096	(35.3)
Moderate, (%)	993	(31.9)
High, (%)	1,020	(32.8)
**BMI–Elevation association**		
BMI < 25.0 and Low elevation, (%)	879	(28.3)
BMI < 25.0 and Moderate elevation, (%)	808	(26.0)
BMI < 25.0 and High elevation, (%)	835	(26.9)
BMI ≥ 25.0 and Low elevation, (%)	217	(7.0)
BMI ≥ 25.0 and Moderate elevation, (%)	185	(6.0)
BMI ≥ 25.0 and High elevation, (%)	185	(6.0)

Notes: Continuous variables are presented as mean (S.D., standard deviation). Discrete variables are presented as frequency (%). MVPA, moderate-to-vigorous physical activity; BMI, body mass index.

**Table 2:** Multivariable logistic regression models.

Variables	CKP		CLBP	
	aOR	95% CI	aOR	95% CI
**BMI**				
<25.0	1.00		1.00	
≥25.0	2.05	1.55–2.71	1.23	0.95–1.59
**Elevation**				
Low	1.00		1.00	
Moderate	0.92	0.68–1.24	0.82	0.63–1.07
High	0.94	0.69–1.29	1.03	0.79–1.34

Notes: CKP, chronic knee pain; CLBP, chronic low back pain; aOR, adjusted odds ratio; 95% CI, 95% confidence interval; MVPA, moderate-to-vigorous physical activity; BMI, body mass index. Multivariable logistic regression analyses were performed after adjustment for age, sex, educational attainment, engaged in farming, self-rated health, depressive symptom, chronic musculoskeletal disorders history, chronic diseases history, and MVPA.

Table 3 shows the associations of BMI and elevation with CKP and CLBP after adjustment for age, sex, educational attainment, engaged in farming, self-rated health, depressive symptom, chronic musculoskeletal disorders history, chronic diseases history, and MVPA. There were statistically significantly higher aORs of CKP for those who were overweight living at low elevation (aOR = 1.90, 95% CI = 1.21–2.98), moderate elevation (aOR = 1.73, 95% CI = 1.05–2.87), and high elevation (aOR = 2.13, 95% CI = 1.31–3.46) than those who were not overweight living at low elevation. However, similar patterns were not observed for CLBP.

**Table 3:** Associations of BMI and elevation with CKP and CLBP.

Variable	CKP		CLBP	
	aOR	95% CI	aOR	95% CI
**BMI—Elevation associations**				
<25.0 and Low	1.00		1.00	
<25.0 and Moderate	0.92	0.65–1.30	0.82	0.61–1.11
<25.0 and High	0.88	0.61–1.27	1.02	0.76–1.38
≥25.0 and Low	1.90	1.21–2.98	1.22	0.80–1.85
≥25.0 and Moderate	1.73	1.05–2.87	0.99	0.62–1.60
≥25.0 and High	2.13	1.31–3.46	1.29	0.82–2.02

Notes: CKP, chronic knee pain; CLBP, chronic low back pain; aOR, adjusted odds ratio; 95% CI, 95% confidence interval; BMI, body mass index; MVPA, moderate-to-vigorous physical activity. Multivariable logistic regression analyses were performed after adjustment for age, sex, educational attainment, engaged in farming, self-rated health, depressive symptom, chronic musculoskeletal disorders history, chronic diseases history, and MVPA.

## 4. Discussion

To the best of our knowledge, no study has examined the associations of overweight and physical environment, measured by elevation, with CKP and CLBP. Considering the context of rural mountainous region in Japan, residents living at higher elevations are more likely to walk on steep slopes, so overweight individuals would have an increased probability of experiencing CKP and CLBP. As expected, our results showed that overweight individuals living at high elevation had a higher aOR of CKP than non-overweight individuals living at low elevation (aOR = 2.13, 95% CI = 1.31–3.46). These results suggest that the overweight–elevation associations may be important in determining CKP in a rural mountainous region. However, similar patterns were not observed for CLBP. 

Our results advance previous debate on the associations between physical environment and chronic musculoskeletal pain. For example, the only study conducted in Japan indicated that there was a significant association between residence on steep slopes and CKP [12]. The limitation of this previous study was that physical environment, *i.e.*, slopes around residence, was assessed by a self-reported questionnaire. Considering that subjective measurement is dependent on respondent’s perception, it would be difficult to design community-based prevention strategy. Our measurement, *i.e.*, elevation, eliminated this problem and it would be helpful to decide target group. In addition, the highlight in this study is to demonstrate the value of environmental factor was different when testing the overweight-elevation association. Although there was no significant association between elevation and CKP, further analysis indicated that overweight individuals living at high elevation had the highest aOR of CKP. 

The mechanisms underlying the associations of overweight and elevation with CKP are most likely of a complex nature and any causal inferences remain to be established. However, as our study population lived in a rural mountainous region, walking up and down on steep slopes may increase mechanical load on the knees [12]. Previous study suggested that living condition could be related musculoskeletal overload in daily activities [9] and occupational activity, *i.e.*, climbing, appears to have an effect on knee osteoarthritis [21]. These findings would support the associations of overweight and elevation with CKP that was observed in this study. In addition, further studies are required to determine whether mechanical load is higher on the knee than on the low back during the various activities described in our setting.

Considering that geographic differences in musculoskeletal pain has been reported [7,8,9,10], community-based prevention activity is needed. As previous study recommended, musculoskeletal pain could be a more socially determined diseases than previously considered [9]. Health professionals should pay attention to these factors in order to formulate efficient health plan for reducing musculoskeletal pain and decide target groups to intervene. For example, in our mountainous region, health education for overweight residents living at higher elevations is needed to promote knowledge of CKP prevention. Moreover, training programs of walking positions on steep slopes could address musculoskeletal pain.

The present study has several strengths. To our knowledge, this is the first study to examine the associations of BMI and elevation with CKP and CLBP. In addition, we studied a random sample of the population and considered a broad range of possible confounders. There are also number of potential limitation. First, misclassification may occur in self-reported assessment data due to recall errors. However, we have no reason to believe that this potential bias differed between residential areas. Second, elevation was used as a proxy for the steepness of their neighborhood. This could lead to misclassification to capture physical environment, *i.e.*, walking in steep slopes. Our findings must be confirmed in other settings to assess this methodology. However, our study is the first of its kind, and could aid in planning community-based interventions. Third, selection bias may exist. The overall response rate in this study was 76.0% and respondents were excluded when they had missing data (51.8% of these were included in the analyses). Finally, the present study used a cross-sectional design that did not allow for the establishment of the temporal order of causality. However, our findings could extend previous discussions about the importance of developing individually-centered prevention strategies with new perspectives focused on physical environment.

## 5. Conclusions

Our results show that specific overweight–elevation associations were observed for CKP in a rural mountainous region. Our results indicate the importance of considering physical environment in establishing prevention strategy for musculoskeletal pain in a rural mountainous region. Further longitudinal research is needed to confirm these new findings and to explore the mechanisms.
